# Vasorelaxant Effect of *Trachelospermi caulis* Extract on Rat Mesenteric Resistance Arteries

**DOI:** 10.3390/molecules27165300

**Published:** 2022-08-19

**Authors:** Chae Eun Haam, Seonhee Byeon, Sooyeon Choi, Eun Yi Oh, Soo-Kyoung Choi, Young-Ho Lee

**Affiliations:** Department of Physiology, Yonsei University College of Medicine, Seoul 03722, Korea

**Keywords:** *Trachelospermi caulis*, vasodilation, mesenteric resistance arteries, vanillin, relaxation, Ca^2+^

## Abstract

Background: *Trachelospermi caulis* (*T. caulis*) has been used as a traditional herbal medicine in Asian countries. Although it is well known that *T. caulis* has beneficial effects, no sufficient research data are available on the cardiovascular effect of *T. caulis*. We investigated whether *T. caulis* extract has vascular effects in rat resistance arteries in this study. Methods: To examine whether *T. caulis* extract affects vascular reactivity, we measured isometric tension of rat mesenteric resistance arteries using a multi-wire myograph system. *T. caulis* extract was administered after arteries were pre-contracted with high K^+^ (70 mM) or phenylephrine (5 µM). Vanillin, a single active component of *T. caulis*, was used to treat mesenteric arteries. Results: *T. caulis* extract caused vascular relaxation in a concentration-dependent manner, which was endothelium-independent. To further identify the mechanism, we incubated the arteries in Ca^2+^-free solution containing high K^+^, followed by a cumulative administration of CaCl_2_ (0.01–2.0 mM) with or without *T. caulis* extract (250 µg/mL). The treatment of *T. caulis* extract decreased contractile responses induced by the addition of Ca^2+^, which suggested that the extracellular Ca^2+^ influx was inhibited by the *T. caulis* extract. Moreover, an active compound of *T. caulis* extract, vanillin, also induced vasodilation in mesenteric resistance arteries. Conclusion: *T. caulis* extract and its active compound, vanillin, concentration-dependently induced vascular relaxation in mesenteric resistance arteries. These results suggest that the administration of *T. caulis* extract could help decrease blood pressure.

## 1. Introduction

Cardiovascular disease (CVD) is the one of the leading causes of morbidity and mortality worldwide. CVD was responsible for 17.3 million deaths worldwide in 2017 which is expected to increase to >23.6 million by 2030 [1,2]. CVD consists of hypertension, heart failure, stroke and a number of vascular and cardiac problems [3]. Hypertension is defined as a systolic blood pressure (SBP) of 130 mmHg or more and a diastolic blood pressure (DBP) of 90 mmHg or more [4].

The arterial system comprises capillaries, arterioles, small arteries, medium-sized arteries and large conduit arteries. Resistance arteries, vessels with lumen diameters measuring <400 μm, are major organs of the cardiovascular system and regulate blood flow and peripheral resistance [5,6]. An increase in vascular resistance caused by the decrease in the internal lumen diameter of the blood vessel is a major cause of elevated blood pressure [7,8]. On the other hand, the relaxation of blood vessels leads to an increase in the lumen diameter of the blood vessels, resulting in an immediate decrease in blood pressure. The relaxation responses of arteries were not only endothelium-dependent but also endothelium-independent. Recently, evidence has been accumulating that endothelium-independent vasodilation is impaired in various vascular beds such as coronary, brachial, forearm and peripheral conduit arteries in patients with cardiovascular diseases and cardiovascular risk factors [9]. In particular, hypertension and diabetes mellitus have been shown to be associated with the impairment of endothelium-independent vasodilation [10,11]. Thus, it is necessary to discover substances that induce endothelium-independent vasodilation as much as substances that cause endothelium-dependent vasodilation.

Although synthetic medications have been widely used to treat and cure patients at various stages of CVD, including hypertension, the adverse effects remain a challenge. Because the treatment of hypertension is continued over a long period of time; therefore, the use of synthetic drugs may result in drug resistance and adverse effects [12]. In addition to the use of synthetic drugs to treat hypertension, the use of a natural product is widely increasing [13,14]. Recently, the use of plant extract has shown a steady growth because of low toxicity and well-established therapy [15]. Various plants used in traditional medicine have been studied for their potential use in the treatment of cardiovascular disease [16]. Vasodilatory effects of medicinal plants have been extensively explored over the last two decades and have proven to be potentially effective for the treatment of CVD in clinics all over the world [17,18,19,20].

*Trachelospermi caulis* (*T. caulis*) belongs to the *Apocynaceae* family, which is the dried leafy stem of *trachelospermum asiaticum* var. *intermedium* [21]. *T. caulis* has been used as traditional herbal medicine to attenuate fever and pain of the knees and loins because of its antipyretic and antinociceptive effects in Asian countries [22]. It is well known that *T. caulis* lowers blood pressure in oriental medicine [23]. Although *T. caulis* has been suggested to provoke beneficial effects, the effect and mechanism of action in cardiovascular system are unknown. Therefore, the aim of our research is to explore the effect of *T. caulis* extract on vascular functionality in resistance arteries and to elucidate the underlying mechanism.

## 2. Results

### 2.1. Effect of T. caulis on Contraction Induced by KCl or PE in Rat Mesenteric Arteries

**Figure 1:**
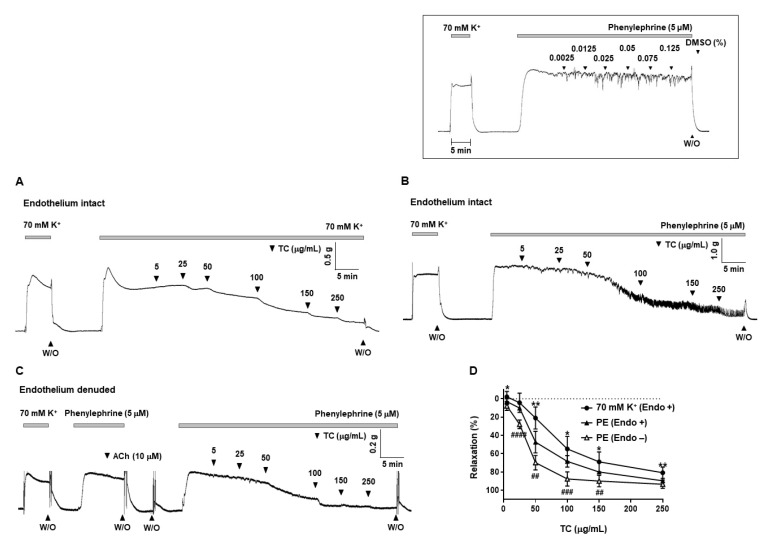
*T. caulis* extract-induced vasodilation in rat mesenteric arteries. (**A**,**B**) Representative traces showing responses to cumulative administration of *T. caulis* extract (5–250 μg/mL) on high K^+^ (**A**) or phenylephrine (**B**)-induced contraction endothelium intact arteries. (**C**) Representative traces showing responses to cumulative administration of *T. caulis* extract (5–250 μg/mL) on phenylephrine-induced contraction in endothelium denuded arteries. (**D**) Statistical analysis of the relaxation response to *T. caulis* extract. Mean ± SD (*n* = 7). Endo + means endothelium-intact arteries and Endo − means endothelium-denuded arteries. * *p* < 0.05 and ** *p* < 0.005 for KCl (Endo +) versus PE (Endo −); ## *p* < 0.005 and ### *p* < 0.001 for PE (Endo +) versus PE (Endo −). Inset, representative trace showing responses to vehicle, DMSO (0.0025–0.125%). (W/O: wash out; TC: *T. caulis* extract; PE: phenylephrine).

Trachelospermi caulis (5–250 μg/mL) induced vascular relaxation in a concentration-dependent manner in endothelium-intact mesenteric arteries pre-contracted with high K^+^ (70 mM) or phenylephrine (PE, 5 μM) and in endothelium-denuded mesenteric arteries pre-contracted with PE (5 μM) as shown in Figure 1. EC_50_ of *T. caulis* is 98.1 μg/mL in the endothelium-intact arteries pre-contracted with high K^+^ (70 mM), 62.36 μg/mL in the endothelium-denuded arteries pre-contracted with PE and 36.61 μg/mL in the endothelium-intact arteries pre-contracted with PE. The maximal relaxation value of *T. caulis*-induced relaxation is 80.73 ± 6.05% in the endothelium-intact arteries pre-contracted with high K^+^, 89.6 ± 2.28% in the endothelium-intact arteries pre-contracted with PE and 93.30 ± 4.46% in the endothelium-denuded arteries pre-contracted with PE. Cumulative administration of vehicle (DMSO, 0.0025–0.125%) did not affect the contraction induced by PE (Figure 1 inset).

### 2.2. T. caulis-Induced Endothelium-Independent Relaxation in Rat Mesenteric Arteries

**Figure 2:**
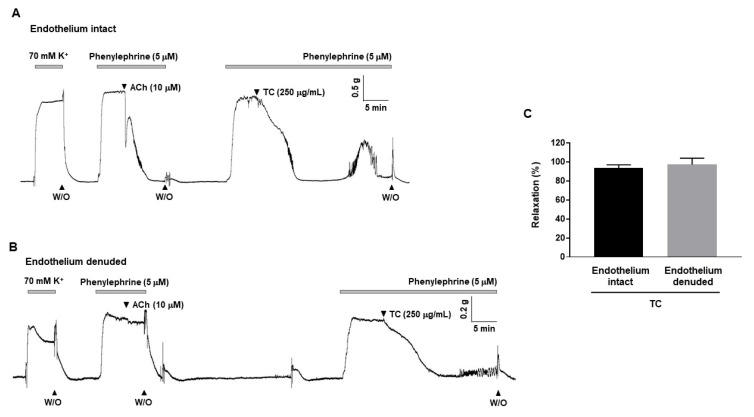
*T. caulis* extract induced endothelium-independent vasodilation in rat mesenteric resistance arteries. (**A**) Representative trace showing *T. caulis* extract-induced vasodilation in the endothelium-intact mesenteric arteries. (**B**) Representative trace showing *T. caulis* extract-induced vasodilation in the endothelium-denuded mesenteric arteries. (**C**) Statistical analysis of *T. caulis*-induced vasodilation. Mean ± SD (*n* = 5). (ACh: acetylcholine; W/O: wash out; TC: *T. caulis* extract).

To explore whether *T. caulis*-induced relaxation is dependent on endothelium. *T. caulis* extract was applied in endothelium-intact (Figure 2A) or endothelium-denuded (Figure 2B) mesenteric arteries. *T. caulis* extract induced vasodilation in the presence and in the absence of the endothelium, and there was no significant difference (93.4 ± 3.56% and 97.17 ± 6.75%, respectively, Figure 2C).

### 2.3. Effects of L-NNA, Indomethacin and ODQ on T. caulis-Induced Vasodilation

**Figure 3:**
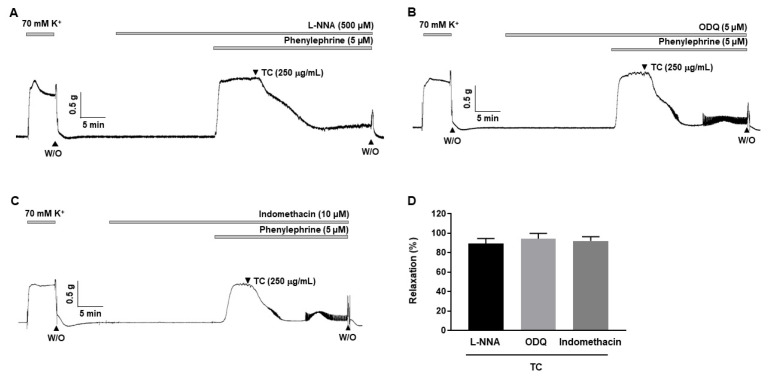
Effect of L-NNA, indomethacin and ODQ on *T. caulis*-induced vasodilation. (**A**–**C**) Representative trace showing *T. caulis* extract-induced vasodilation in the presence of L-NNA (**A**), ODQ (**B**) and indomethacin (**C**). (**D**) Statistical analysis of the relaxation response of *T. caulis* extract in the presence of L-NNA, ODQ and indomethacin. Relaxation of arteries is expressed as the percentage of the contraction induced by PE (5 μΜ). Mean ± SD (*n* = 5). (TC: *T. caulis* extract; L-NNA: Nω-Nitro-L-arginine; ODQ: 1H-[1,2,4]-oxadiazolo-[4,3-α]-quinoxalin-1-one; W/O: wash out).

To investigate the involvement of the nitric oxide (NO)/cyclic guanosine monophosphate (cGMP) and cyclooxygenase (COX)/prostacyclin (PGI_2_) pathways in *T. caulis*-induced vasodilation, arteries were incubated for 20 min with endothelial nitric oxide (eNOS) inhibitor, Nω-Nitro-L-arginine (L-NNA, 500 μM), or soluble guanylyl cyclase (sGC) inhibitor, 1H-(1,2,4)oxadiazolo[4,3-a]quinoxalin-1-one (ODQ, 5 μM), or COX inhibitor, indomethacin (10 μM), before being contracted with PE (5 μM). The relaxation responses of *T. caulis* were 89.39 ± 5.12%, 94.41 ± 5.41% and 92.03 ± 4.45% in the presence of L-NNA, ODQ and indomethacin, respectively (Figure 3).

### 2.4. Effects of K^+^ Channel Blockers on T. caulis Extract-Induced Vascular Relaxation

**Figure 4:**
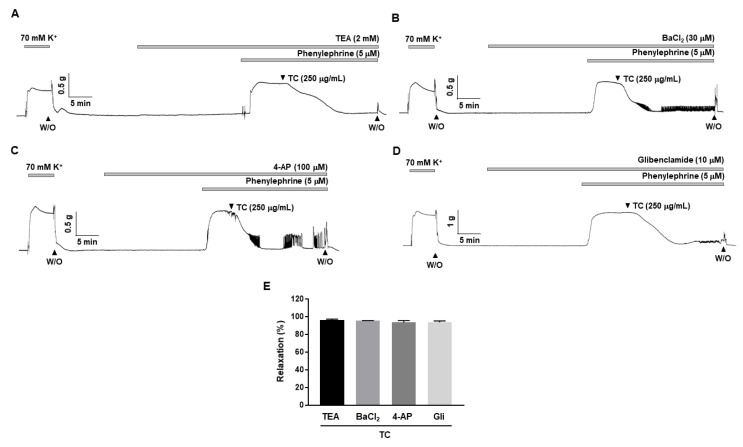
Effect of K^+^ channel blockers on *T. caulis* extract-induced vasodilation. (**A**–**D**) Effects of *T. caulis* extract in the mesenteric arteries pre-contracted with PE (5 μΜ) in the presence of TEA (**A**), BaCl_2_ (**B**), 4-AP (**C**) or glibenclamide (**D**). (**E**) Statistical analysis of the relaxation response of *T. caulis* extract in the presence of K^+^ blockers. Relaxation of arteries is expressed as the percentage of the contraction induced by PE (5 μΜ). Mean ± SD (*n* = 7). (TC: *T. caulis* extract; TEA: tetraethylammoni-um; Gli: glibenclamide; 4-AP: 4-aminopyridine; W/O: wash out).

To determine whether K^+^ channels are involved in *T. caulis*-induced relaxation, non-selective K^+^ channel blocker, tetraethylammonium (TEA, 2 mM) or inward rectifier K^+^ channel blocker, BaCl_2_ (30 μM) or voltage-dependent K^+^ channel blocker, 4-aminopyridine (4-AP, 100 μM) or ATP-sensitive K^+^ channel blocker, glibenclamide (10 μM), were pre-treated 20 min before being contracted with PE (5 μM). The relaxation responses of *T. caulis* were 96.23 ± 2.72%, 95.26 ± 0.27%, 93.44 ± 2.10% and 93.51 ± 1.62%, in the presence of TEA, BaCl_2_, 4-AP and glibenclamide, respectively (Figure 4).

### 2.5. Effect of T. caulis on Extracellular Ca^2+^-Induced Contraction

**Figure 5:**
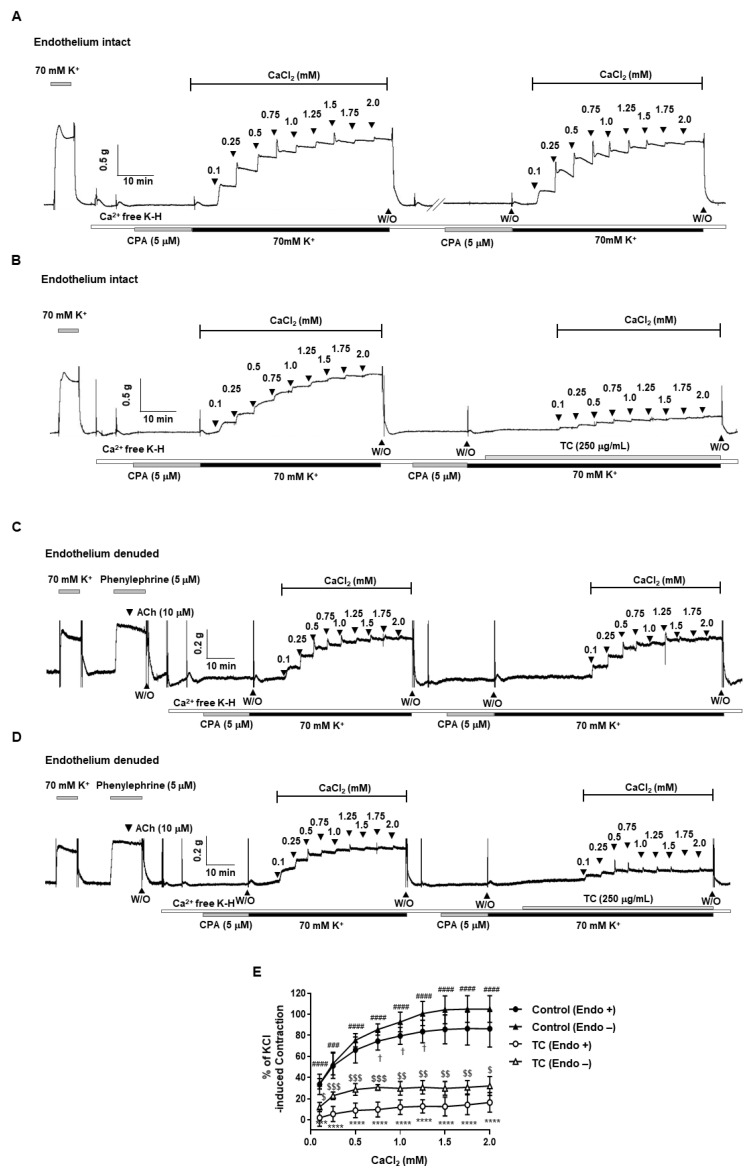
*T. caulis* extract inhibited extracellular Ca^2+^-induced vasoconstriction. (**A**,**C**) Representative traces showing the contraction responses by repeated addition of Ca^2+^ are not changed in endothelium-intact (**A**) and endothelium-denuded (**C**) arteries. (**B**,**D**) Representative traces showing the effect of *T. caulis* in the mesenteric arteries treated with cumulative addition of CaCl_2_ (0.1–2.0 mM) in endothelium-intact (**B**) and endothelium-denuded (**D**) arteries. (**E**) Statistical analysis of contraction induced by CaCl_2_ in the mesenteric arteries with or without *T. caulis*. Mean ± SD (*n* = 7). *** *p* < 0.001 and **** *p* < 0.0001 for control (Endo +) versus TC (Endo +); ### *p* < 0.001 and #### *p* < 0.0001 for control (Endo −) versus TC (Endo −); † *p* < 0.05 for control (Endo +) versus control (Endo −); $ *p* < 0.05, $$ *p* < 0.01, and $$$ *p* < 0.001 for TC (Endo +) versus TC (Endo −). Endo + means endothelium-intact arteries and Endo − means endothelium-denuded arteries. (W/O: wash out; TC: *T. caulis* extract).

To identify whether the vasodilatory effect of *T. caulis* depends on the inhibition of extracellular Ca^2+^ influx, the mesenteric arteries were incubated in a Ca^2+^-free solution containing sarcoplasmic reticulum Ca^2+^-ATPase (SERCA) inhibitor, cyclopiazonic acid (CPA, 5 μΜ) and KCl (70 mM), and then CaCl_2_ was added by concentration (0.1–2.0 mM) to increase the Ca^2+^ concentration in the arteries. Before treating the arteries with *T. caulis*, it was confirmed that the contraction responses caused by the repeated addition of Ca^2+^ were not changed in endothelium-intact and endothelium-denuded mesenteric arteries (Figure 5A,C). Pre-treatment of *T. caulis* significantly reduced the contractile responses induced by the cumulative addition of Ca^2+^ in endothelium-intact and endothelium-denuded mesenteric arteries (Figure 5B,D).

### 2.6. Effect of T. caulis on the BAY K8644-Induced Contraction

**Figure 6:**
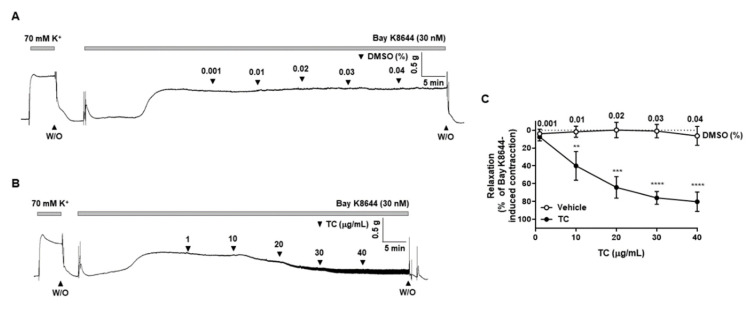
*T. caulis* extract reduced contraction induced by L-type voltage-gated calcium channel activation. (**A**) Representative trace showing the contraction response was not changed by treatment of vehicle (DMSO). (**B**) Representative trace showing the effect of *T. caulis* on the mesenteric arteries pre-constricted by BAY K8644. (**C**) Statistical analysis of relaxation induced by TC extract in the mesenteric arteries pre-constricted by BAY K8644. Mean ± SD (*n* = 7). ** *p* < 0.01, *** *p* < 0.001, and **** *p* < 0.0001 for DMSO versus TC (W/O: wash out; TC: *T. caulis* extract).

To confirm that voltage-gated calcium channel is involved in the *T. caulis* extract-induced vasodilation, arteries were pre-treated with L-type voltage-gated calcium channel activator, BAY K8644, and then *T. caulis* extract was administered in the mesenteric arteries. Treatment of vehicle (DMSO 0.001–0.04%) did not affect BAY K8644-induced contraction (Figure 6A). *T. caulis* extract concentration dependently induced vascular relaxation (Figure 6B).

### 2.7. Effects of Vanillin, a Single Active Compound of T. caulis, on Mesenteric Arteries

**Figure 7:**
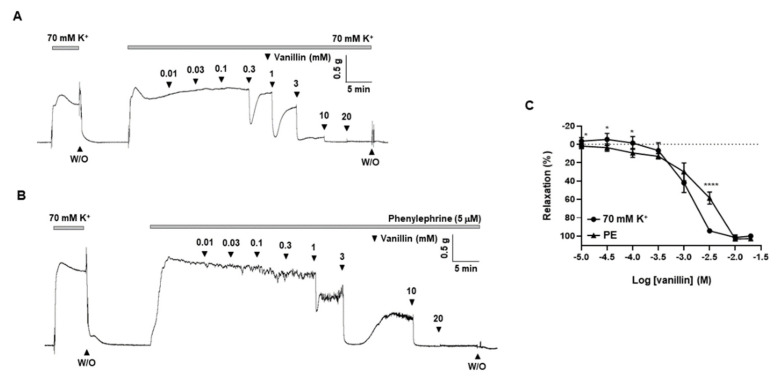
Vanillin-induced vasodilation in rat mesenteric resistance arteries. (**A**,**B**) Representative traces showing responses to cumulative administration of vanillin (0.01–20 mM) on high-K^+^ (**A**) or PE (**B**)-induced contraction. (**C**) Statistical analysis of the relaxation response to vanillin. Mean ± SD (*n* = 7). * *p* < 0.05 and **** *p* < 0.0001 for KCl versus PE (W/O: wash out).

To support the findings about vascular effect of *T. caulis*, we further investigated whether single active compounds of *T. caulis* exert similar effect as *T. caulis* extract. The gas chromatogram of the compounds identified in the extract of *T. caulis* is shown in Figure S1 of supplementary materials. The identities of 12 compounds were determined along with their retention time (Table S1 of supplementary materials). The compounds identified based on the gas chromatography–mass spectrometry (GC/MS) analysis include butanoic acid (butyric acid), cyclobutanol, 3-nitropropanoic acid, furan-2-carbaldehyde (furfural), 4-hydroxy-3-methoxybenzaldehyde (vanillin), 4-hydroxy-3-methoxybenzaldehyde,(1R,2S,3S,4R,5R)-6,8-dioxabicyclo[3.2.1]octane-2,3,4-triol, 3-hydroxy-4-methoxybenzoic acid, 3,4,5,6-tetrahydroxy-2-methoxyhexanal, 3,4,5,6-tetrahydroxy-2-methoxyhexanal,(3S,4S,5R,6S)-3-methoxy-6-(methoxymethyl)oxane-2,4,5-triol, hexanoic acid, 1-(2,6-dihydroxy-4-methoxyphenyl)butan-1-one and benzoic acid. Among these candidate single compounds, we found that 4-hydroxy-3-methoxybenzaldehyde (vanillin) has a vascular relaxation effect. We observed that vanillin (0.01–20 mM) induced vasodilation in a concentration-dependent manner in rat mesenteric arteries pre-contracted with high-K^+^ solution (70 mM) or PE (5 μM). (Figure 7). EC_50_ of vanillin is 1.1 mM in the mesenteric arteries pre-contracted with high K^+^ (70 mM) and 1.9 mM in the arteries pre-contracted with PE. We also tested vascular effects of two more single compounds, butyric acid and furfural, in mesenteric resistance arteries. However, butyric acid did not induce significant vasodilation (Figure S2 of supplementary materials). As shown in Figure S3 of supplementary materials, furfural induced vasodilation in a very high concentration (10 mM).

## 3. Discussion

The aim of the present study was to examine the direct effect of *T. caulis* extract on the vascular functionality in resistance arteries and to determine the underlying mechanism. We demonstrated that *T. caulis* reduced contraction induced by KCl or PE in rat mesenteric resistance arteries. *T. caulis* concentration-dependently induced vascular relaxation in the presence and absence of the endothelium. Additionally, the pre-treatment of L-NNA, ODQ and indomethacin did not affect the vasodilatory effect of *T. caulis*, which indicates that *T. caulis*-induced relaxation is not related with the NO pathway. The K^+^ channel blockers, TEA, BaCl_2_, 4-AP and glibenclamide, did not affect the *T. caulis*-induced relaxation either. *T. caulis* inhibited extracellular Ca^2+^-induced vasoconstriction responses in the mesenteric arteries. In addition, one of the active compounds of *T. caulis*, vanillin, also has a similar relaxation effect.

*Trachelospermi caulis* is well-known herb that is used to alleviate swelling from sore throats and carbuncles, as well as to lower fever from muscular contracture and rheumatoid arthritis [24]. Although, beneficial effects of *T. caulis* have been well-reported, no sufficient data are available on the cardiovascular effect of *T. caulis*. This is the first study that presents the effect of *T. caulis* on the vascular responses in rat mesenteric resistance arteries.

The vascular endothelium is a single layer lining the luminal surface of vessels. In response to various stimuli, endothelium releases vasoactive substances such as nitric oxide (NO), prostacyclin (prostaglandin I_2_; PGI_2_), endothelium-derived hyperpolarizing factor (EDHF), thromboxane (TXA2) and endothelin-1 (ET-1) [25]. Among these substances, it is well known that NO, PGI_2_ and EDHF induce vasodilation [26]. In the present study, we investigated whether *T. caulis* causes vascular relaxation through endothelial cells. We found that *T. caulis* induced vasodilation in the presence and in the absence of endothelium, and there was no significant difference. These finding suggested that *T. caulis*-induced vasodilation is endothelium-independent. In order to confirm that *T. caulis*-induced vascular relaxation occurs endothelium independent, we performed experiments using various inhibitors such as L-NNA, ODQ and indomethacin. After NO is generated by nitric oxide synthase (NOS) in endothelium, it diffuses into smooth muscle cells and activates sGC to increase intracellular cyclic guanosine monophosphate (cGMP) concentration, resulting in relaxation [27]. We found that there was no significant difference in the vasodilatory effect of pre-treatment with the L-NNA and ODQ. These results show that *T. caulis* does not relax blood vessels through the NO-cGMP pathway. Another vasodilator factors released from endothelial cells, PGI_2_, is produced by cyclooxygenase (COX) [28]. In the case of pre-treatment with indomethacin, a non-selective COX inhibitor, there was no significant difference in the vasodilation effect of *T. caulis* compared to the control. These results indicate that the vasodilatory effects of *T. caulis* are not related to PGI_2_. Taken together, *T. caulis* does not cause a relaxation effect through endothelium.

Since it has been confirmed that *T. caulis*-induced vasodilation is endothelium-independent, we next investigated whether *T. caulis* causes a vascular relaxation by acting on smooth muscle directly. Vascular smooth muscle relaxation is initiated by decrease in intracellular Ca^2+^, which results from reduction of extracellular Ca^2+^ influx or Ca^2+^ releases from intracellular store (SR) [29]. Activation of K^+^ channel induces K^+^ efflux which causes membrane hyperpolarization. Membrane hyperpolarization contributes to closure of VDCC to block the influx of extracellular Ca^2+^, which induces relaxation of the smooth muscle cells [30]. In the previous study, it has been reported that K^+^ channel is involved in the vasodilatory effect of plant extract in mesenteric arteries [31]. In this study, we treated mesenteric arteries with various types of K^+^ channel blockers such as TEA, BaCl_2_, 4-AP and glibenclamide. Pre-treatment with K^+^ channel blockers did not affect the vasodilatory effect of *T. caulis*. These results indicate that the vasodilatory effect of *T. caulis* was not induced by the activation of K^+^ channels.

Next, we examined whether *T. caulis* caused relaxation through reduction of Ca^2+^ influx. Because the blocking the Ca^2+^ channels did not induce contraction of the arteries, the relaxation effect failed to be tested in the presence of Ca^2+^ channel blockers. Thus, we used an alternative method to test the effect of *T. caulis*. The mesenteric arteries were incubated in the Ca^2+^ free K-H solution containing CPA, which is the state that intracellular Ca^2+^ is depleted. Then, the 70 mM K^+^ is administered to enable the opening of VDCC. The cumulative addition of Ca^2+^ induced contractile responses in mesenteric arteries, which is reduced by administration of *T. caulis*. To confirm that VDCC is inhibited by treatment of *T. caulis* extract, we used VDCC activator, BAY K8644. Since treatment of BAY K8644 alone did not cause stable contraction, arteries were incubated with K-H solution containing 15 mM of K^+^ which create an environment where VDCC could be opened. *T. caulis* extract concentration dependently induced vascular relaxation in the arteries pre-contracted with BAY K8644 (Figure 6B). These results suggest that extracellular Ca^2+^ influx was inhibited by treatment of *T. caulis* extract.

Although we discovered vasodilatory effect of *T. caulis*, further study was required to confirm whether single active compound of *T. caulis* extract also induces vascular relaxation in mesenteric arteries. Among the several compounds identified based on gas chromatography–mass spectrometry (GC/MS), we the tested effect of 4-hydroxy-3-methoxybenzaldehyde (vanillin), butyric acid and furfural on the mesenteric arteries. We found that butyric acid did not induce significant relaxation responses in the arteries (Figure S2 of supplementary materials) and only a high concentration of furfural could exert a vasodilatory effect on the mesenteric arteries (Figure S3 of supplementary materials). Interestingly, vanillin has a potent vasodilatory effect, such as *T. caulis* extract (Figure 7), on mesenteric arteries. This result is in accordance with a previous study which reported that vanillin induced relaxation in porcine coronary and basilar arteries [32]. Vanillin is a small molecule that is commonly used as flavoring agents or as additives by the food and cosmetics industries. It is considered that vanillin has various biological functions such as anti-inflammatory, antioxidative and neuroprotective functions [33,34]. In the present study, vanillin induced vascular relaxation in a concentration-dependent manner in mesenteric arteries pre-contracted with PE and high K^+^. These results support our findings that *T. caulis* induces vasodilation in mesenteric resistance arteries.

Plants contain a variety of metabolites including polyphenols, catechins, flavonoids, alkaloid and many volatile components [35]. Some of these metabolites have been suggested to have cardio-protective and antihypertensive effects [16]. The present study suggests the potential use of *T. caulis* extracts as antihypertensive agents by showing that the *T. caulis* extract significantly relaxes resistance arteries.

## 4. Materials and Methods

### 4.1. Animals and Tissue Preparation

All experiments were performed according to the Guide for the Care and Use of Laboratory Animals published by the US National Institutes of Health (NIH publication No. 85-23, 2011) and were approved by the Ethics Committee and the Institutional Animal Care and Use Committee of Yonsei University, College of Medicine (Approval number: 2021-0058). In this experiment, 8–11-week-old male Sprague Dawley rats were used. After being sacrificed, the mesenteric arteries were rapidly dissected and placed in an ice-cold Krebs–Henseleit (K-H) solution (composition (mM): NaCl 119, KCl 4.6, MgSO_4_ 1.2, KH_2_PO_4_ 1.2, CaCl_2_ 2.5, NaHCO_3_ 25 and glucose 11.1) bubbled with 95% O_2_ and 5% CO_2_. Adipose and connective tissue were removed from the mesenteric arteries using a microscope (model SZ-40, Olympus, Shinjuku, Tokyo, Japan). The 2nd branches of mesenteric arteries (250–300 μm) were cut into 2–3 mm-long sections and used in this experiment. If necessary, endothelium was removed by gently rubbing using thin forceps.

### 4.2. Preparation of T. caulis Extract

The plant extract (CW01-037) used in this research was obtained from the Korea Plant Extract Bank at the Korea Research Institute of Bioscience and Biotechnology (Daejeon, Korea). The plant (103 g) dried in the shade and powdered was added to 1 L of distilled water and heat-extracted for 150 min at 100 °C in an extractor (DW-290, DAEWOONG ELECTRONIC APPIIANCE). After filtration and drying under freeze drying using a freeze dryer (Clean Vac 8, HANIL SCIENCE Co., Ltd.), the yield of the *T. caulis* extract was 8.3% (8.55 g) of the plant powder and was dissolved in 5% of dimethyl sulfoxide (DMSO).

### 4.3. Measurement of Isometric Tension in Mesenteric Arteries

Isolated segments were mounted in wire myography (model 620M, Danish Myotechnology, Aarhus, Denmark) for the recording of isometric tension. Arteries were bathed in 37 °C K-H solution, constantly bubbled with 95% O_2_ and 5% CO_2_. Vessels were equilibrated for 20 min and stretched to their optimal resting tension ~4 mN. Contractility of the vessel was evaluated by incubating KCl (70 mM) 3 times. The response was recorded by stabilizing the vessel by contracting the arteries to KCl (70 mM) or PE (5 μM), followed by a cumulative addition of extract (5–250 μg/mL) or vehicle (DMSO, 0.00025–0.125%). To investigate the mechanism of vascular relaxation of the aqueous extract of *T. caulis*, L-NNA, indomethacin, ODQ, TEA, BaCl_2_, glibenclamide or 4-AP were pre-treated for 20 min, and then the relaxation response of the *T. caulis* (250 μg/mL) to phenylephrine (5 μM) contraction was recorded. An experiment was conducted to determine the effect of Ca^2+^ on vascular relaxation when the *T. caulis* (250 μg/mL) is treated. CPA (5 μM) was treated in Ca^2+^-free solution to remove both intracellular and extracellular calcium. After replacing the solution with Ca^2+^-free K-H solution containing 70 mM of KCl and CPA to Ca^2+^-free K-H solution containing 70 mM of KCl, changes in contraction were recorded by increasing the concentration of CaCl_2_ on arteries. The extract was then pre-treated in the same arteries for 20 min and the changes in contractility in the same experiment were compared with those before pre-treatment with the *T. caulis*. The CaCl_2_-induced contraction was calculated as the percentage of maximum contraction recorded from the KCl contraction. In addition, some arteries were pre-contracted by BAY K8644 (30 nM) in K-H solution containing 15 mM KCl to investigate *T. caulis*-induced relaxation.

### 4.4. Chemicals and Reagents

Phenylephrine hydrochloride (PE), ACh, L-NNA, ODQ, TEA, BaCl_2_, 4-AP, butyric acid, furfural and vanillin were purchased from Sigma-Aldrich (St. Louis, MO, USA). Indomethacin was obtained from Calbiochem (Darmstadt, Germany). Glibenclamide was purchased from Tocris Bioscience (Bristol, UK). CPA was obtained from Enzo Life Sciences (Farmingdale, NY, USA).

### 4.5. Statistical Analysis

All values are expressed as mean ± standard deviations. One-way or two-way ANOVA was used to compare the groups when appropriate. Comparisons between groups were performed with t-tests when the ANOVA test was statistically significant. For all experiments measuring diameter, the n-values mean the number of vessels derived from each different animal. Values of * *p* < 0.05 were considered statistically significant. Differences between specified groups were analyzed using the Student’s *t* test (2-tailed) to compare the two groups, with * *p* < 0.05 considered statistically significant.

## 5. Conclusions

In the present study, for the first time, we discovered that *T. caulis* extract induced vascular relaxation in rat mesenteric resistance arteries. The vasodilatory effect of *T. caulis* was endothelium-independent and the inhibition of extracellular Ca^2+^ influx was related to *T. caulis* extract-induced vascular relaxation. Our results suggest that *T. caulis* could be a valuable herbal resource in future research and in the treatment of cardiovascular diseases.

## Data Availability

Data are available upon appropriate requests.

## Supplementary Materials

The following supporting information can be downloaded at: https://www.mdpi.com/article/10.3390/molecules27165300/s1, Figure S1: Gas Chromatography–Mass Spectrometry (GC/MS) of the Compounds in *T. caulis* Extract; Figure S2: Effect of butyric acid in mesenteric arteries; Figure S3: Effect of furfural in mesenteric arteries; Table S1: Bioactive compounds detected in *T. caulis* extract.

## References

[1] Virani S.S., Alonso A., Benjamin E.J., Bittencourt M.S., Callaway C.W., Carson A.P., Chamberlain A.M., Chang A.R., Cheng S., Delling F.N. (2020). Heart disease and stroke statistics-2020 update: A report from the american heart association. Circulation.

[2] Zhou D., Xi B., Zhao M., Wang L., Veeranki S.P. (2018). Uncontrolled hypertension increases risk of all-cause and cardiovascular disease mortality in us adults: The nhanes iii linked mortality study. Sci. Rep..

[3] Masaebi F., Salehi M., Kazemi M., Vahabi N., Looha M.A., Zayeri F. (2021). Trend analysis of disability adjusted life years due to cardiovascular diseases: Results from the global burden of disease study 2019. BMC Public Health.

[4] de Lima R.S., Silva J.C.S., Lima C.T., de Souza L.E., da Silva M.B., Baladi M.G., Irigoyen M.C., Lacchini S. (2019). Proinflammatory role of angiotensin ii in the aorta of normotensive mice. Biomed. Res. Int..

[5] Christensen K.L., Mulvany M.J. (2001). Location of resistance arteries. J. Vasc. Res..

[6] Touyz R.M., Alves-Lopes R., Rios F.J., Camargo L.L., Anagnostopoulou A., Arner A., Montezano A.C. (2018). Vascular smooth muscle contraction in hypertension. Cardiovasc. Res..

[7] Dunn W.R., Gardiner S.M. (1995). Structural and functional properties of isolated, pressurized, mesenteric resistance arteries from a vasopressin-deficient rat model of genetic hypertension. Hypertension.

[8] Schiffrin E.L. (1992). Reactivity of small blood-vessels in hypertension—relation with structural-changes—state-of-the-art lecture. Hypertension.

[9] Maruhashi T., Kihara Y., Higashi Y. (2018). Assessment of endothelium-independent vasodilation: From methodology to clinical perspectives. J. Hypertens..

[10] Maruhashi T., Soga J., Fujimura N., Idei N., Mikami S., Iwamoto Y., Kajikawa M., Matsumoto T., Hidaka T., Kihara Y. (2013). Nitroglycerine-induced vasodilation for assessment of vascular function: A comparison with flow-mediated vasodilation. Arterioscler. Thromb. Vasc. Biol..

[11] Adams M.R., Robinson J., McCredie R., Seale J.P., Sorensen K.E., Deanfield J.E., Celermajer D.S. (1998). Smooth muscle dysfunction occurs independently of impaired endothelium-dependent dilation in adults at risk of atherosclerosis. J. Am. Coll. Cardiol..

[12] Handler J. (2005). Quality of life and antihypertensive drug therapy. J. Clin. Hypertens..

[13] Alaerts G., Merino-Arevalo M., Dumarey M., Dejaegher B., Noppe N., Matthijs N., Smeyers-Verbeke J., Vander Heyden Y. (2010). Exploratory analysis of chromatographic fingerprints to distinguish rhizoma chuanxiong and rhizoma ligustici. J. Chromatogr. A.

[14] Newman D.J., Cragg G.M. (2020). Natural products as sources of new drugs over the nearly four decades from 01/1981 to 09/2019. J. Nat. Prod..

[15] Xia M., Qian L., Zhou X., Gao Q., Bruce I.C., Xia Q. (2008). Endothelium-independent relaxation and contraction of rat aorta induced by ethyl acetate extract from leaves of morus alba (l.). J. Ethnopharmacol..

[16] Ibarra-Alvarado C., Rojas A., Mendoza S., Bah M., Gutierrez D.M., Hernandez-Sandoval L., Martinez M. (2010). Vasoactive and antioxidant activities of plants used in mexican traditional medicine for the treatment of cardiovascular diseases. Pharm. Biol..

[17] Luna-Vazquez F.J., Ibarra-Alvarado C., Rojas-Molina A., Rojas-Molina I., Zavala-Sanchez M.A. (2013). Vasodilator compounds derived from plants and their mechanisms of action. Molecules.

[18] Pandey K.B., Rizvi S.I. (2009). Plant polyphenols as dietary antioxidants in human health and disease. Oxidative Med. Cell. Longev..

[19] Shaito A., Thuan D.T.B., Phu H.T., Nguyen T.H.D., Hasan H., Halabi S., Abdelhady S., Nasrallah G.K., Eid A.H., Pintus G. (2020). Herbal medicine for cardiovascular diseases: Efficacy, mechanisms, and safety. Front. Pharmacol..

[20] Liu C., Huang Y. (2016). Chinese herbal medicine on cardiovascular diseases and the mechanisms of action. Front. Pharmacol..

[21] Shin H.S., Bae M.J., Jung S.Y., See H.J., Kim Y.T., Do J.R., Back S.Y., Choi S.W., Shon D.H. (2015). Enhancing effect of trachelogenin from trachelospermi caulis extract on intestinal barrier function. Biol. Pharm. Bull..

[22] Liu X.T., Wang X.G., Yang Y., Xu R., Meng F.H., Yu N.J., Zhao Y.M. (2015). Qualitative and quantitative analysis of lignan constituents in caulis trachelospermi by hplc-qtof-ms and hplc-uv. Molecules.

[23] Hempen C.H., Fischer T. (2001). A Materia Medica for Chinese Medicine: Plants, Minerals, and Animal Products.

[24] Lee M.H., Lee J.M., Jun S.H., Ha C.G., Lee S.H., Kim N.W., Lee J.H., Ko N.Y., Mun S.H., Park S.H. (2007). In-vitro and in-vivo anti-inflammatory action of the ethanol extract of trachelospermi caulis. J. Pharm. Pharmacol..

[25] Rajendran P., Rengarajan T., Thangavel J., Nishigaki Y., Sakthisekaran D., Sethi G., Nishigaki I. (2013). The vascular endothelium and human diseases. Int. J. Biol. Sci..

[26] Rubanyi G.M. (1991). Endothelium-derived relaxing and contracting factors. J. Cell. Biochem..

[27] Munzel T., Daiber A., Ullrich V., Mulsch A. (2005). Vascular consequences of endothelial nitric oxide synthase uncoupling for the activity and expression of the soluble guanylyl cyclase and the cgmp-dependent protein kinase. Arterioscler. Thromb. Vasc. Biol..

[28] Sellers R.S., Radi Z.A., Khan N.K. (2010). Pathophysiology of cyclooxygenases in cardiovascular homeostasis. Vet. Pathol..

[29] Leloup A.J.A., Van Hove C.E., De Moudt S., De Meyer G.R.Y., De Keulenaer G.W., Fransen P. (2019). Vascular smooth muscle cell contraction and relaxation in the isolated aorta: A critical regulator of large artery compliance. Physiol. Rep..

[30] Gollasch M., Ried C., Bychkov R., Luft F.C., Haller H. (1996). K+ currents in human coronary artery vascular smooth muscle cells. Circ. Res..

[31] Kwon Y., Haam C.E., Byeon S., Choi S.J., Shin D.H., Choi S.K., Lee Y.H. (2020). Vasodilatory Effect of Phellinus linteus Extract in Rat Mesenteric Arteries. Molecules.

[32] Raffai G., Khang G., Vanhoutte P.M. (2015). Vanillin and vanillin analogs relax porcine coronary and basilar arteries by inhibiting l-type ca2+ channels. J. Pharmacol. Exp. Ther..

[33] Kim H.J., Hwang I.K., Won M.H. (2007). Vanillin, 4-hydroxybenzyl aldehyde and 4-hydroxybenzyl alcohol prevent hippocampal ca1 cell death following global ischemia. Brain Res..

[34] Wang P., Li C., Liao G., Huang Y., Lv X., Liu X., Chen W., Zhang L. (2022). Vanillin attenuates proinflammatory factors in a tmcao mouse model via inhibition of tlr4/nf-kb signaling pathway. Neuroscience.

[35] Sultana S., Asif H.M. (2017). Review: Medicinal plants combating against hypertension: A green antihypertensive approach. Pak. J. Pharm. Sci..

