# Supplementary material for: Vasorelaxant Effect of Trachelospermi caulis Extract on Rat Mesenteric Resistance Arteries
Source: Molecules. 2022 Aug 19;27(16):5300. doi: 10.3390/molecules27165300 (PMC9413539; doi:10.3390/molecules27165300)

Supplementary Materials

Supplementary Figure S1. Gas Chromatography–Mass Spectrometry (GC/MS) of the Compounds in *T. caulis* Extract

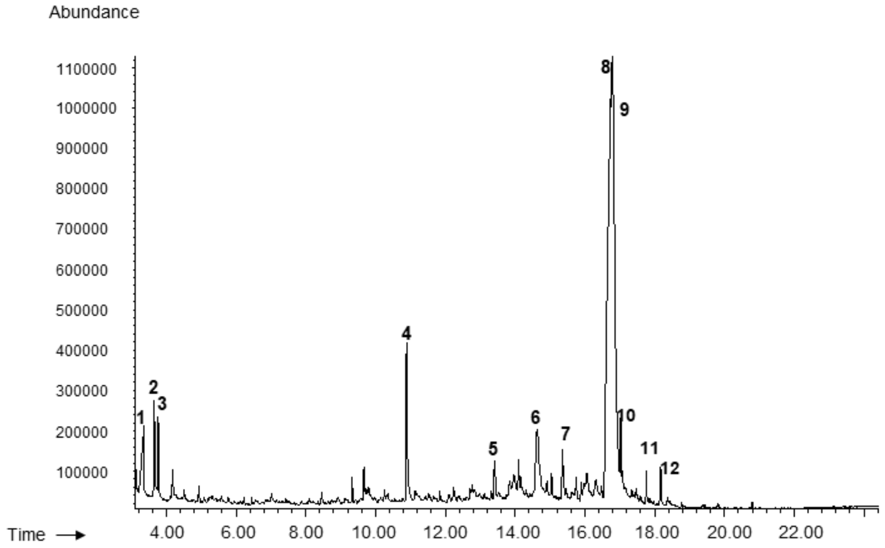

Supplementary Table S1. Bioactive compounds detected in *T. caulis* extract.

Peak	RT (min)	Compound	Peak Area (%)
1	3.35	butanoic acid	3.62
2	3.65	cyclobutanol	2.64
3	3.77	3-nitropropanoic acid	1.64
4	10.89	furan-2-carbaldehyde	5.13
5	13.41	4-hydroxy-3-methoxybenzaldehyde	1.32
6	14.64	(1R,2S,3S,4R,5R)-6,8-dioxabicyclo[3.2.1]octane-2,3,4-triol	6.57
7	15.37	3-hydroxy-4-methoxybenzoic acid	1.53
8	16.74	3,4,5,6-tetrahydroxy-2-methoxyhexanal	30.64
9	16.79	(3S,4S,5R,6S)-3-methoxy-6-(methoxymethyl)oxane-2,4,5-triol	38.51
10	17.03	hexanoic acid	1.38
11	17.76	1-(2,6-dihydroxy-4-methoxyphenyl)butan-1-one	0.56
12	18.18	benzoic acid	1.07

Supplementary Figure S2. Effect of butyric acid in mesenteric arteries.

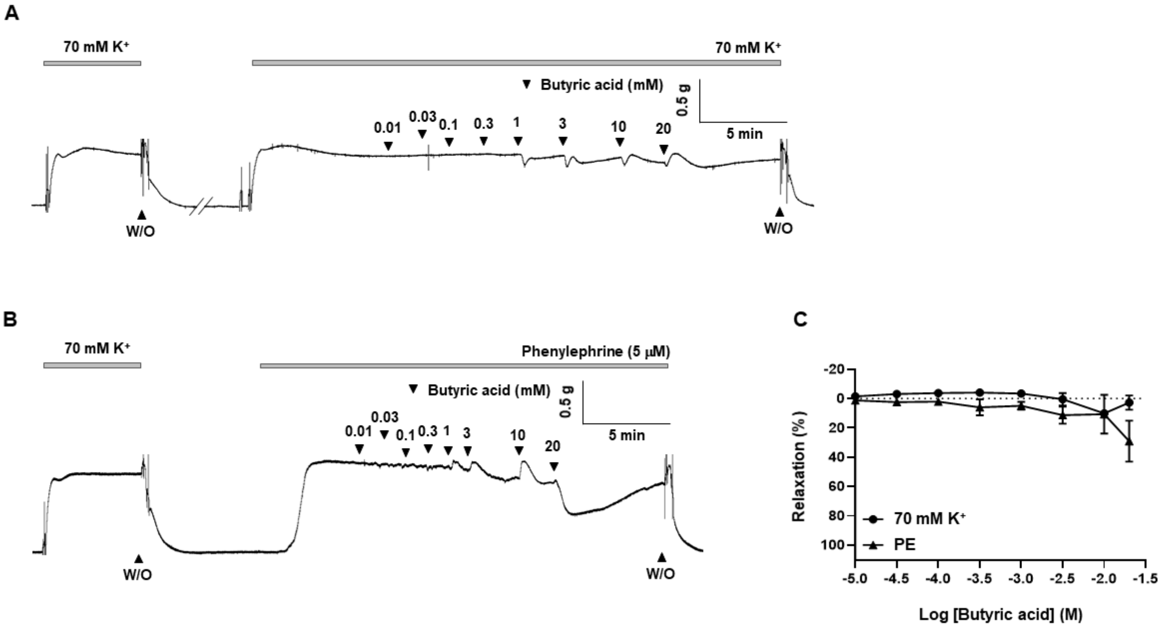

Supplementary Figure S3. Effect of furfural in mesenteric arteries.

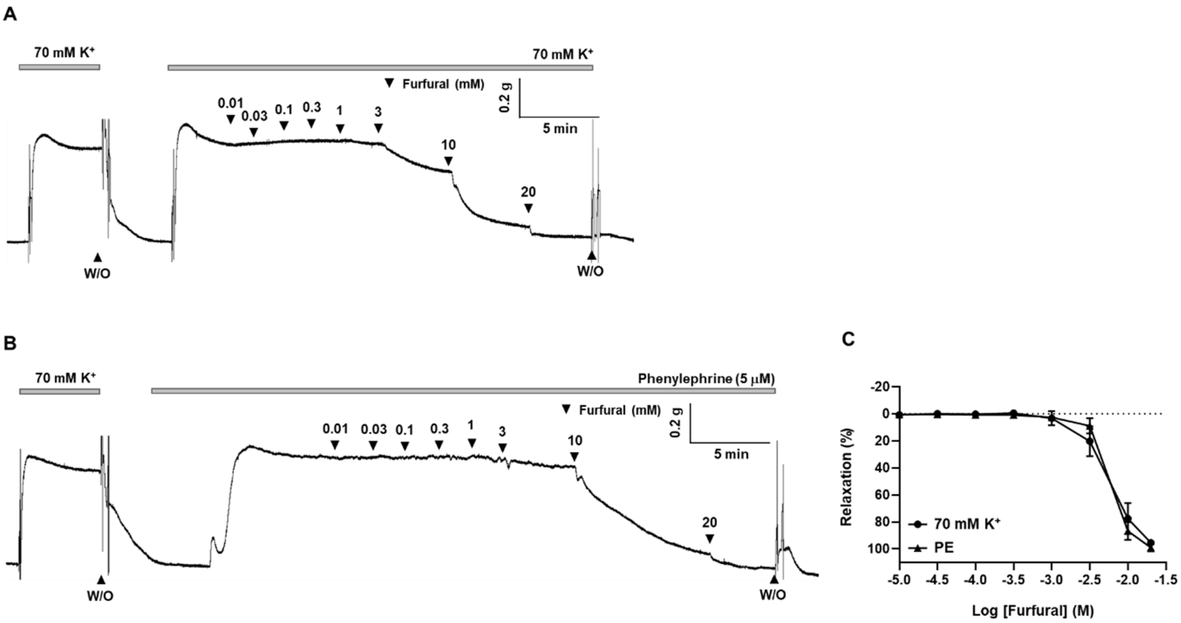

Supplement: Supplementary file 1 [file molecules-27-05300-s001.zip › molecules-1741977-supplementary.pdf]
